# Evaluation of bone mineralization in former preterm born children: Phalangeal quantitative ultrasound cannot replace dual-energy X-ray absorptiometry

**DOI:** 10.1016/j.bonr.2018.01.004

**Published:** 2018-01-28

**Authors:** Carmen M.T. Lageweg, Mayke E. van der Putten, Johannes B. van Goudoever, Ton Feuth, Martin Gotthardt, Arno F.J. van Heijst, Viola Christmann

**Affiliations:** a Department of Pediatrics, Subdivision of Neonatology, Radboudumc Amalia Children's Hospital, Radboud University Medical Center, Nijmegen, The Netherlands; b Department of Pediatrics, Subdivision of Neonatology, Maastricht University Medical Center, Maastricht, The Netherlands; c Department of Pediatrics, VU University Medical Center Amsterdam, Amsterdam, The Netherlands; d Departement of Pediatrics, Emma Children's Hospital – AMC Amsterdam, Amsterdam, The Netherlands; e Department for Health Evidence, Radboud Institute for Health Science, Subdivision of Biostatistics, Radboud university medical center, Nijmegen, The Netherlands; f Department of Radiology and Nuclear Medicine, Radboud university medical center, Nijmegen, The Netherlands

**Keywords:** AD-SoS, amplitude dependent speed of sound, *BMC*, bone mineral content, *BMD*, bone mineral density, *BTT*, bone transit time, *DXA*, dual-energy X-ray absorptiometry-scan, *pQUS*, phalangeal quantitative ultrasound, *QUS*, quantitative ultrasound, *SOS*, speed of sound, *ISCD*, International Society of Clinical Densitometry, Bone development, Children, Correlation, Dual-energy X-ray absorptiometry-scan, Speed of sound, Ultrasound

## Abstract

**Background:**

Preterm infants are at risk of impaired bone health in later life. Dual-energy X-ray absorptiometry-scan (DXA) is the gold standard to determine bone mineralization. Phalangeal quantitative ultrasound (pQUS) is an alternative technique that is inexpensive, easy to use and radiation-free. The aim of this study was to investigate whether both techniques reveal equivalent results.

**Materials and methods:**

Sixty former preterm infants (31 boys; 29 girls) received a DXA and pQUS at age 9 to 10 years. DXA measured bone mineral content (BMC) and bone mineral density (BMD) for total body and lumbar spine (L1-4), while pQUS measured the amplitude dependent speed of sound (AD-SoS) and bone transit time (BTT) at metacarpals II-IV providing continuous values and *Z*-scores based on age and sex. Four statistical methods evaluated the association between both techniques: Pearson's correlation coefficients, partial correlation coefficients adjusted for gestational age, height and BMI, Bland-Altman analysis and cross tabulation.

**Results:**

Both techniques showed a statistically significant weak correlation for continuous values as well as *Z*-scores (0.291–0.462, p < 0.05). Boys had significant and relatively high correlations (0.468–0.585, p < 0.05). In comparison, the correlations for girls were not significant. Correlation coefficients further decreased while calculating the partial correlations. The Bland-Altman plots showed poor agreement. Sensitivity ranged from 33% to 92% and specificity from 16% to 68%. Positive and negative predictive values ranged from 4% to 38% and 82% to 97%, respectively.

**Conclusions:**

We found statistically significant weak correlations and poor agreement between DXA and pQUS measurements. DXA is not equivalent to pQUS and therefore not replaceable by this technique in former preterm born children at the age of 9 to 10 years.

## Highlights

• DXA is the golden standard to determine bone mineralization, while Quantitative ultrasound is a radiation-free alternative.
• Four statistical tests were used to investigate the equivalence of both methods.
• Comparison of results of 60 preterm born children at age 9 to 10 years found poor agreement between both methods.
• There was a discrepancy in differentiating the same children with normal or reduced bone mineralization.
• Quantitative ultrasound cannot replace the DXA scan for determination of bone mineralization in preterm born children.

## 1 Introduction

Bone development is one of the key processes during fetal, neonatal and infant development (Schoenau et al., 2004). Mineralization of bone mainly starts during the third trimester of pregnancy based on active placental transfer of calcium and phosphorus to the fetus. Up to 80% of the body calcium of a term infant is accrued during the last trimester (Kovacs, 2014). Preterm infants miss out the active fetal bone development and therefore are at risk of reduced bone mineralization and development of osteopenia (Harrison et al., 2008). Inadequate bone mineralization is seen as a risk factor for the development of osteoporosis in later life, which is an important cause of morbidity and mortality in elderly people and a considerable factor of healthcare expenditure (Kannus et al., 1999; Javaid and Cooper, 2002; Leppälä et al., 1999). The peak bone mass is attained before skeletal maturity (Bonjour et al., 1991). Any factor that influences the acquisition of peak bone mass may represent a mechanism to affect later osteoporosis risk. The evaluation of bone development in preterm born children is relevant for the determination of the individual health risk as well as the evaluation of medical treatment that aimed at improvement in bone development.

Currently, there are two techniques available to determine bone mineralization, either dual-energy X-ray absorptiometry-scan (DXA) or quantitative ultrasound (QUS). DXA is the most commonly used technique for assessing bone mineralization in children and adolescents (Wren and Gilsanz, 2006). Although DXA is a non-invasive and standardized method, it is not available for all medical centers and it uses a low amount of radiation. In recent years, QUS has been proposed as an alternative method to replace DXA for the evaluation of bone status, especially since it is relatively inexpensive, fast, easy to use, portable and radiation-free (Baroncelli, 2008; Gianni et al., 2008; Tansug et al., 2011; Tuna et al., 2008).

Studies investigating the association between the measurements of DXA and QUS revealed inconsistent results. While a number of studies showed a significant positive correlation between DXA and QUS (Van Rijn et al., 2000; Di Mase et al., 2012; Falcini et al., 2000; Gonçalves et al., 2014; Hartman et al., 2004a; Pluskiewicz et al., 2002; Sani et al., 2011; Sundberg et al., 1998; Xu et al., 2014; Bąk-Drabik et al., 2016; Catalano et al., 2017; Olszynski et al., 2016; Zuckerman-Levin et al., 2007; Mora et al., 2009; Weeks et al., 2016; Halaba et al., 2005; Catalano et al., 2013), others found a discrepancy between the measurements of the two methods (Gianni et al., 2008; Halaba et al., 2005; Chong et al., 2015; Christoforidis et al., 2010; Christoforidis et al., 2011; Hartman et al., 2004b; Williams et al., 2012; Alwis et al., 2010). This could be a result of the different QUS measurement sites or different patient categories investigated. Only a limited number of studies used the phalangeal QUS (Di Mase et al., 2012; Gonçalves et al., 2014; Pluskiewicz et al., 2002; Bąk-Drabik et al., 2016; Catalano et al., 2017; Olszynski et al., 2016; Halaba et al., 2005; Catalano et al., 2013) and only one study looked at the specific group of former preterm born children (Gianni et al., 2008).

The aim of this study was to investigate whether the measurements of dual-energy X-ray absorptiometry scan (DXA) and phalangeal quantitative ultrasound (pQUS) performed in preterm born children aging from 9 to 10 years reveal comparable results. We hypothesized that both techniques were equivalent in diagnosing the state of bone mineralization. Equivalent results would mean that the pQUS could replace the DXA for evaluation bone mineralization as a diagnostic tool.

## 2 Materials and methods

### 2.1 Study design

This study was a cross-sectional study using the data collection of the study “Long-term follow up of growth and bone mineralization of former preterm infants” (FoBoMin). This study was approved by the Ethics committee (CMO nr 2013/594) of the Radboud University Medical Center. Informed consent was obtained from all parents after approval by the local ethics committee.

### 2.2 Study population and procedure

The study included 60 former preterm infants at the age of 9 to 10 years. All subjects participated in the FoBoMin-study. This long-term follow-up study evaluated two cohorts of very preterm infants with a birth weight below 1500 g and gestational age < 34 weeks. The cohorts differed by nutritional intake during the first two weeks of life. The second cohort received higher intake of protein, energy as well as calcium and phosphate. This was associated with improved weight gain during the early postnatal period (Christmann et al., 2013). The aim of the FoBoMin-study was to compare long-term growth and bone mineralization in relation to early nutritional intake in preterm born children at age 9 to 10 years. All participants of the studies were evaluated by DXA and pQUS. The measurements were performed on the same day for the individual participant. Four statistical methods were used to compare both methods.

### 2.3 Measurement instruments and variables

Bone mineralization of the total body and lumbar spine (L1-L4) was determined using the QDR Discovery A S/N 85606 (Hologic, Inc., USA). According to the International Society for Clinical Densitometry (ISCD), the lumbar spine (L1-L4) and whole body scan are the preferred skeletal sites for measurement in children (Lewiecki et al., 2008). The measurements of the DXA were analyzed using the APEX-system software version 13.3. The DXA uses a low dose of radiation depending on measurement site. The effective dose, reflecting the real radiation risk for children of 10 years old, for the whole body is 4.8 μSv and for the lumbar spine 7.1 μSv (Blake et al., 2006). According to the ‘Rijksinstituut voor Volksgezondheid en Milieu’ (RIVM) the yearly averaged ambient dose equivalent rate for the NMR station in the area of Nijmegen is 74 nSv/h (Knetsch, 2013), resulting in a daily exposure in Nijmegen of 1.78 μSv. Therefore, the radiation dose of DXA can be regarded as very low and is negligible. Results of the DXA were expressed as Bone Mineral Content (BMC; g), Bone Mineral Density (BMD; g/cm^2^), representing the ratio between BMC and bone area (cm^2^), and *Z*-scores, representing the number of standard deviations above or below the mean for the patients' sex and age. The *Z*-scores were calculated by the DXA software on the basis of reference values for sex and age obtained from a large U.S. population provided by the manufacturer. The *Z*-scores of the whole body were calculated using the reference data of the National Health and Nutrition Examination Survey (NHANES, 2008) (Kelly et al., 2009), while lumbar spine *Z*-scores were based on the reference data of the Bone Mineral Density in Childhood Study (BMDCS) (Zemel et al., 2011). A Z-score less than or equal to −2.0 SD is considered to indicate ‘low bone mineral status’ (Lewiecki et al., 2008).

The quantitative ultrasound (pQUS) was performed on the second to the fifth metacarpals of the phalangeal bones using a DBM Sonic Bone Profiler (IGEA, Carpi, Italy). The mean value of the measurements per person was calculated. The transmitter of the pQUS generated a sound frequency of 1.25 MHz. This technique measured the amplitude dependent speed of sound (AD-SoS) and bone transit time (BTT), which were both expressed in continuous values and in *Z*-scores. The AD-SoS (m/s) was the ultrasound velocity inside the finger and was derived from the measurement of the time interval between emission and reception of the ultrasound signal, considering the first signal with a minimum amplitude of 2 mV at the receiver probe. The BTT (μsec) reflected the bone characteristics without the interference of the soft tissue by calculating the difference between transmission time in soft tissue and bone and transmission time in soft tissue (Di Mase et al., 2012). The *Z*-scores were determined on the basis of the reference values related to sex and age (AD-SoS Z score (age); BTT- Z-score (age)) or sex and height (AD-SoS Z score (height); BTT Z-score (height)). The Z-scores were obtained from a large Italian population provided by the manufacturer (Baroncelli et al., 2006).

Additionally, age, sex, gestational age at birth, weight, height, BMI and pubertal development were recorded. Weight (kg) was measured using an electronic digital scale (SECA MOD701) to the nearest 0.1 kg. Height (cm) was determined using a vertical stadiometer (SECA MOD240) to the nearest 0.1 cm. Body mass index (BMI; kg/m^2^) was calculated by dividing weight (kg) by the square of height (m^2^). Pubertal development was self-assessed from pictures showing the different Tanner stages (Tye, 2016). The children were asked to indicate which picture most resembled their current appearance.

### 2.4 Statistical analysis

The statistical analysis was performed using the Statistical Package for the Social Sciences (IBM SPSS Inc., Chicago, IL, USA, version 22.0). All results were expressed as mean ± SD. Four statistical methods were used for the analysis of the association between pQUS and DXA. First, the Pearson's correlation coefficients (r) were calculated for evaluation of the correlation between continuous values as well as the *Z*-scores of DXA and pQUS. The correlation coefficients were determined for every outcome for the total group as well as for boys and girls separately. Secondly, the partial correlation coefficients were determined to correct for possible confounders on the original correlation between DXA and pQUS. Possible confounders of bone development, such as age, sex, gestational age, weight, height, BMI and Tanner stages at follow-up were included in the analysis. Only three of these confounders, namely gestational age, height and BMI were used to calculate partial correlation coefficients, because of the limited number of participants in this study. The three confounders were chosen based on calculating whether they correlated significantly with DXA and pQUS measurements. Thirdly, a Bland-Altman analysis was performed to evaluate the agreement between both techniques using the *Z*-scores of either DXA and pQUS. Plots were created with the mean of two Z-scores within the same subject resulting from the two techniques on the horizontal axis and the difference of the Z-scores on the vertical axis. Finally, a cross tabulation was performed and the sensitivity, specificity as well as positive and negative predictive values were calculated, where DXA was considered as the gold standard. In agreement with the ISCD, a DXA *Z*-score less than or equal to −2.0 SDS should be considered as low bone mineralization (Lewiecki et al., 2008). A Z-score between −1.0 and −2.0 was considered as reduced (Aceto et al., 2014) and a *Z*-score above −1.0 SDS is normal. For the current study a cut-off value of −1.0 SDS was used for the assessment of low or normal bone mineralization, in the absence of participants with a Z-score less than −2.0.

A two tailed p-value of <0.05 was considered statistically significant.

## 3 Results

### 3.1 Patient characteristics

The baseline characteristics of the participants (total group as well as boys and girls separately) are presented in Table 1. Anthropometric characteristics, gestational age at birth and pQUS measurements at follow-up were comparable between boys and girls. No statistically significant differences were found.

**Table 1:** Baseline characteristics and bone parameters for participants.

	Total (n = 60)	Boys (n = 31)	Girls (n = 29)	p-Value
Age (y)	10.0 ± 0.5	9.9 ± 0.5	10.0 ± 0.6	0.78
Gestational age (weeks)	29.4 ± 1.7	29.2 ± 1.4	29.6 ± 1.9	0.27
Weight (kg)	31.1 ± 5.5	30.9 ± 4.4	31.3 ± 6.5	0.79
Height (cm)	139.3 ± 5.9	139.8 ± 6.3	138.7 ± 5.4	0.45
BMI (kg/m^2^)	16.0 ± 2.3	15.7 ± 1.4	16.2 ± 3.0	0.43
Tanner stage pubic hair (n/stage)	46/I; 13/II; 1/III	25/I; 6/II	21/I; 7/II; 1/III	0.64
Tanner stage mammae	22/I; 6/II, 1/III	–	22/I; 6/II, 1/III	–


DXA measurements	Total (n = 60)	Boys (n = 31)	Girls (n = 29)	p-Value
Whole body BMC (g)	1123.5 ± 145.0	1136.8 ± 156.4	1109.3 ± 133.0	0.47
Whole body BMD (g/cm^2^)	0.851 ± 0.064	0.859 ± 0.061	0.842 ± 0.066	0.29
Whole body *Z*-score	0.4 ± 1.0	0.5 ± 1.0	0.3 ± 1.0	0.49
Lumbar spine BMC (g)	24.5 ± 4.2	24.2 ± 4.5	24.8 ± 4.0	0.57
Lumbar spine BMD (g/cm^2^)	0.601 ± 0.084	0.580 ± 0.085	0.623 ± 0.078	0.05
Lumbar spine *Z*-score	−0.1 ± 1.1	−0.2 ± 1.3	0.1 ± 1.0	0.32


pQUS measurements	Total (n = 60)	Boys (n = 31)	Girls (n = 29)	p-Value
AD-SoS (m/s)	1830.9 ± 72.7	1814.2 ± 75.8	1848.8 ± 65.8	0.07
AD- SoS *Z*-score (age)	−2.3 ± 1.7	−2.7 ± 1.9	−2.0 ± 1.5	0.15
AD-SoS Z-score (height)	−2.2 ± 1.8	−2.5 ± 2.1	−1.9 ± 1.6	0.23
BTT (μsec)	0.8 ± 0.3	0.8 ± 0.3	0.7 ± 0.3	0.57
BTT *Z*-score (age)	−0.9 ± 1.5	−0.9 ± 1.7	−0.8 ± 1.2	0.77
BTT *Z*-score (height)	−1.0 ± 1.4	−1.0 ± 1.6	−1.0 ± 1.3	0.95

All data are presented as mean ± SD. *P*-values of the difference between boys and girls were calculated using an unpaired *t*-test.

AD-SoS, amplitude-dependent speed of sound; BTT, bone transit time; BMC, bone mineral content; BMD, bone mineral density; *Z*-score (age), Z-score adjusted for sex and age; *Z*-score (height), Z-score adjusted for sex and height.

### 3.2 Correlation

Table 2 presents the correlation coefficients of DXA and pQUS measurements for the continuous values. The correlation coefficients between the DXA and both pQUS measurements (BTT; AD-SoS) showed statistical significance, though the r value was low. The correlation coefficients between DXA and BTT were higher, ranging from 0.341 to 0.462 (p < 0.05), compared to correlation coefficients between DXA and AD-SoS, ranging from 0.291 to 0.345 (p < 0.05). In comparison, boys showed a statistically significant and slightly higher correlation, which was not found for girls (boys: 0.468–0.585, p < 0.05 versus girls: 0.008–0.335, p > 0.05). Nevertheless, the differences found between boys and girls, calculated with the Fisher's r-to-Z transformation, were not statistically significant, except for lumbar spine BMD and AD-SoS (p = 0.039).

**Table 2:** Pearson correlation coefficients between continuous results of pQUS and DXA measurements.

		DXA											
		Total (*n* = 60)				Boys (*n* = 31)				Girls (*n* = 29)			
		Whole body BMC	Whole body BMD	Lumbar spine BMC	Lumbar spine BMD	Whole body BMC	Whole body BMD	Lumbar spine BMC	Lumbar spine BMD	Whole body BMC	Whole body BMD	Lumbar spine BMC	Lumbar spine BMD
*pQUS*	AD-SoS	**0.325 (0.011)**	**0.313 (0.015)**	**0.291 (0.024)**	**0.345 (0.007)**	**0.474 (0.007)**	**0.474 (0.007)**	**0.479 (0.006)**	**0.518 (0.003)**	0.195 (0.312)	0.236 (0.218)	0.008 (0.969)	0.011 (0.956)
	BTT	**0.462 (0.000)**	**0.393 (0.002)**	**0.399 (0.002)**	**0.341 (0.008)**	**0.585 (0.001)**	**0.525 (0.002)**	**0.566 (0.001)**	**0.468 (0.008)**	0.335 (0.076)	0.273 (0.152)	0.250 (0.192)	0.284 (0.135)

All data are presented as: correlation coefficient and in brackets the p-value.

pQUS, phalangeal quantitative ultrasound; DXA, dual-energy X-ray absorptiometry; AD-SoS, amplitude-dependent speed of sound; BTT, bone transit time; BMC, bone mineral content; BMD, bone mineral density.

Table 3 presents the correlation coefficients for the *Z*-scores. AD-SoS Z-score (age) and BTT Z-score (age) showed a statistically significant but weak correlation with DXA *Z*-scores (0.327–0.401, p ≤ 0.05). The correlation coefficients for BTT Z-scores (age) were higher than those for AD-SoS Z-scores (age). Since the Z-scores (height) showed no statistically significant correlation coefficients, they were not further evaluated. In comparison, the *Z*-scores (age) of boys showed a statistically significant correlation coefficient in contrast to girls (boys: 0.436–0.520, p < 0.05 versus girls: −0.026–0.274, p > 0.05). In general, the difference found between boys and girls, calculated with the Fisher's r-to-Z transformation, was not statistically significant, except for lumbar spine *Z*-score and AD-SoS Z-score (age) (p = 0.027).

**Table 3:** Pearson correlation coefficients between pQUS *Z*-scores and DXA Z-scores.

		DXA					
		Total (*n* = 60)		Boys (*n* = 31)		Girls (*n* = 29)	
		Whole body *Z*-score	Lumbar spine Z-score	Whole body Z-score	Lumbar spine Z-score	Whole body Z-score	Lumbar spine Z-score
pQUS	AD-SoS Z-score (age)	**0.327 (0.011)**	**0.335 (0.009)**	**0.511 (0.003)**	**0.520 (0.003)**	0.131 (0.498)	−0.026 (0.894)
	AD-SoS Z-score (height)	0.244 (0.060)	0.225 (0.084)	**0.419 (0.019)**	**0.384 (0.033)**	0.037 (0.850)	−0.105 (0.589)
	BTT *Z*-score (age)	**0.401 (0.001)**	**0.367 (0.004)**	**0.499 (0.004)**	**0.436 (0.014)**	0.274 (0.150)	0.234 (0.222)
	BTT *Z*-score (height)	0.188 (0.150)	0.225 (0.084)	**0.413 (0.021)**	**0.376 (0.037)**	−0.113 (0.559)	−0.009 (0.962)

All data are presented as: correlation coefficient and in brackets the p-value.

pQUS, phalangeal quantitative ultrasound; DXA, dual-energy x-ray absorptiometry; AD-SoS, amplitude-dependent speed of sound; BTT, bone transit time; *Z*-score (age), *Z*-score adjusted for sex and age; Z-score (height), Z-score adjusted for sex and height.

**Fig. 1:**
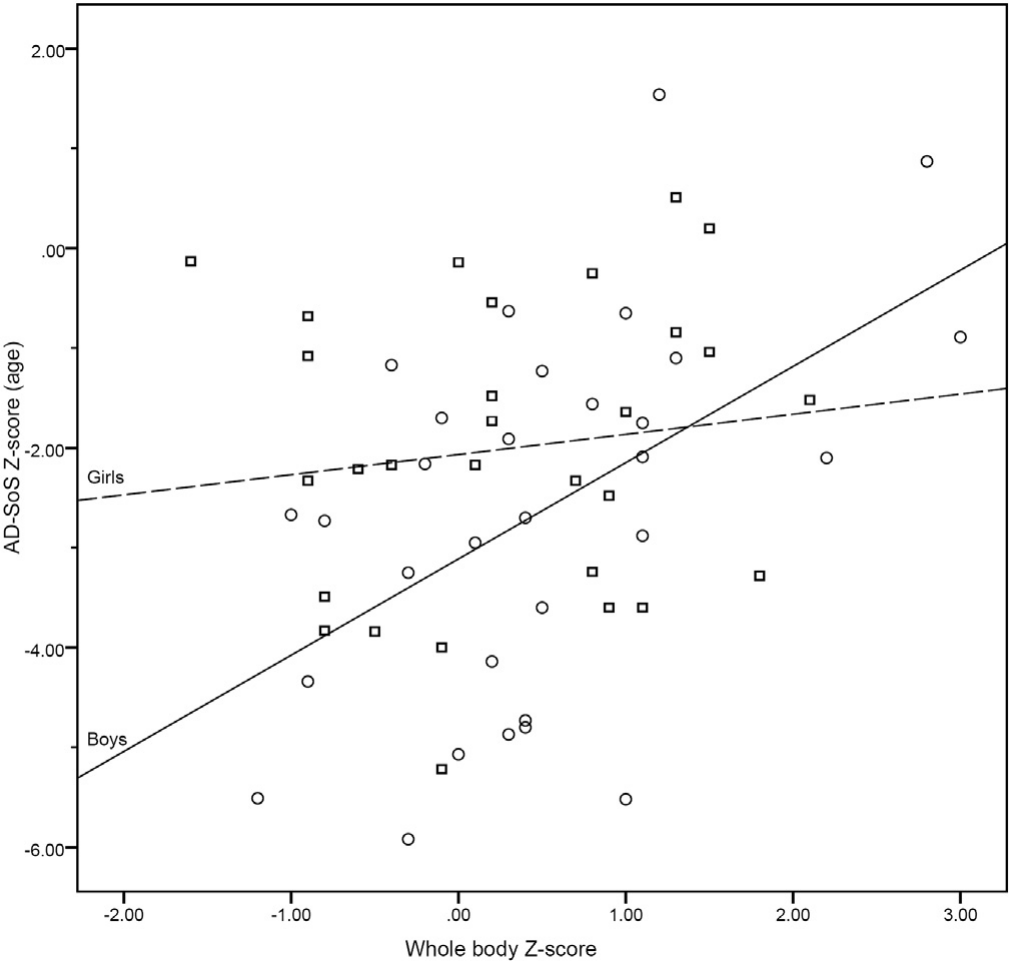
Overlay scatterplot. Scatterplot for the correlation coefficients between AD-SoS *Z*-score (age) and whole body Z-score for boys and girls. The circles and the continuous line represent boys. The squares and the broken line represent girls.

As an example, Fig. 1 illustrates an overlay scatterplot of the correlation coefficients between the AD-SoS *Z*-score (age) and the whole body Z-score for boys and girls. Other pQUS and DXA measurements revealed comparable scatterplots.

### 3.3 Partial correlation

Table 4 presents the original correlation coefficients and the partial correlation coefficients adjusted for gestational age, height and BMI for the continuous values and the *Z*-scores (age). The adjustment for the three confounders induced a further decrease of the correlation coefficients. On average, the remaining coefficients, although significant, were very weak.

**Table 4:** Correlation coefficients and partial correlation coefficients (adjusted for gestational age, height and BMI) of continuous variables and Z-scores of DXA and pQUS.

		AD-SoS	BTT	AD-SoS Z-score (age)	BTT Z-score (age)
Whole body BMC	Correlation coefficient (r)	0.325	0.462	–	–
	Partial correlation coefficient	0.215	0.267	–	–
	p-Value partial coefficient	0.004	0.000	–	–
Whole body BMD	Correlation coefficient (r)	0.313	0.393	–	–
	Partial correlation coefficient	0.220	0.236	–	–
	p-Value partial coefficient	0.012	0.009	–	–
Lumbar spine BMC	Correlation coefficient (r)	0.291	0.399	–	–
	Partial correlation coefficient	0.191	0.241	–	–
	p-Value partial coefficient	0.024	0.005	–	–
Lumbar spine BMD	Correlation coefficient (r)	0.345	0.341	–	–
	Partial correlation coefficient	0.280	0.249	–	–
	p-Value partial coefficient	0.012	0.031	–	–
Whole body *Z*-score	Correlation coefficient (r)	–	–	0.327	0.401
	Partial correlation coefficient	–	–	0.238	0.294
	p-Value partial coefficient	–	–	0.012	0.002
Lumbar spine *Z*-score	Correlation coefficient (r)	–	–	0.335	0.367
	Partial correlation coefficient	–	–	0.274	0.295
	p-Value partial coefficient	–	–	0.019	0.011

AD-SoS, amplitude-dependent speed of sound; BTT, bone transit time; BMC, bone mineral content; BMD, bone mineral density; *Z*-score (age), Z-score adjusted for sex and age.

### 3.4 Agreement

**Fig. 2:**
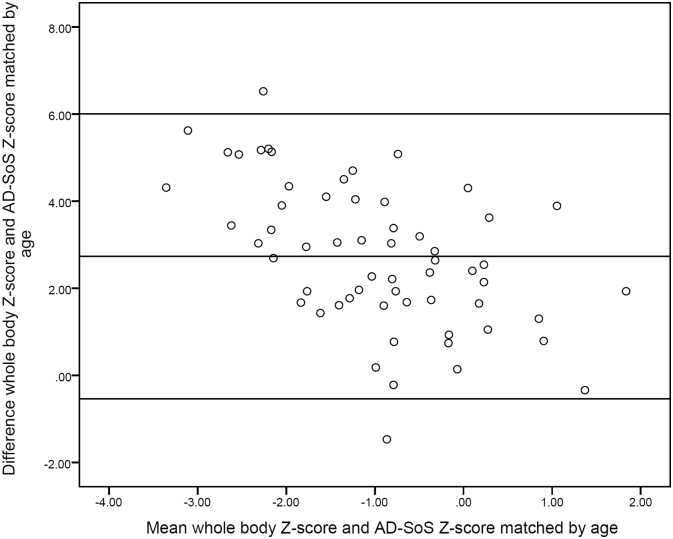
Bland-Altman plot. The Bland- Altman plot presents the agreement between whole body Z-score measured by dual-energy X-ray absorptiometry and AD-SoS Z-score (age) measured by phalangeal quantitative ultrasound. The middle horizontal line represents the mean difference of the Z-score; the upper and lower horizontal lines represent the 95% limits-of-agreement.

The agreement was considered for all parameters. Only the Bland-Altman plot of the whole body *Z*-score and the AD-SoS Z-score (age) will be presented, because the other plots showed comparable results (Fig. 2). The agreement between the two techniques was low, based on the following results. First, the mean difference between the *Z*-scores as presented in Fig. 2 was 2.73 and thereby significantly different from zero determined using a paired *t*-test (*p*-value: 0.011). Thereby, the 95%-limits of agreement in this figure had a large interval between −0.54 and 6.00. Lastly, the plot showed that the differences of the two *Z*-scores (whole body Z-score minus AD-SoS Z-score (age)) were negatively dependent on the mean. This means that the difference between DXA and pQUS Z-scores increased with a lower mean Z-score, leading to an increasing disagreement between the two techniques while assessing bone mineralization for lower *Z*-scores.

### 3.5 Cross tabulation

Cross tabulation was performed for all combinations of pQUS and DXA measurements. The number of pQUS measurements with a *Z*-score below −1.0 SDS was higher compared to DXA. The sensitivity for all measurements ranged from 33% to 92%. The specificity ranged from 16% to 68%. The positive and negative predictive values ranged from 4% to 38% and 82% to 97%, respectively.

As an example, Table 5 shows a cross table for the BTT *Z*-score (age) and the lumbar spine Z-score. This table revealed the best agreement of all measurements, but the specificity, sensitivity, positive predictive value and negative predictive value in general, were low. The sensitivity and specificity for BTT Z-score (age) in comparison with lumbar spine Z-score were 69% and 68%, respectively, and for the positive and negative predictive value this was 38% and 89%, respectively. Overall, we found a large discrepancy between the two methods for discriminating a patient with a normal or reduced bone mineralization.

**Table 5:** Cross table of the BTT *Z*-score (age) measured by pQUS and the lumbar spine Z-score measured by DXA.

	DXA – Lumbar spine Z-score	
pQUS – BTT *Z*-score (age)	*Z*-score ≤ −1	Z-score > −1
Z-score ≤ −1	9	15
Z-score > −1	4	32

Cut-off value = −1.0 SDS (≤−1: reduced bone mineralization; >−1: normal bone mineralization).

pQUS, phalangeal quantitative ultrasound; DXA, dual-energy X-ray absorptiometry; BTT, bone transit time; *Z*-score (age), Z-score adjusted for sex and age.

## 4 Discussion

This study evaluated two different diagnostic techniques for bone development in former preterm born children, who are at risk for impaired bone mineralization. Four statistical tests showed that the results of dual-energy X-ray absorptiometry scan (DXA) and phalangeal quantitative ultrasound (pQUS) had a significant weak correlation that further decreased after adjustment for confounders. In addition, there was a low agreement between the two techniques and a discrepancy in differentiating the same children with normal or reduced bone mineralization.

The correlation coefficients were calculated for the continuous values as well as for the standard deviation scores (*Z*-scores) based on reference data. According to Baroncelli (2008) the *Z*-score is the more appropriate value to express bone mineralization in children. The DXA Z-scores were available adjusted for sex and age, while the pQUS presented two types of Z-scores, either adjusted for sex and age (*Z*-score (age)) or sex and height (Z-score (height)). In our study the continuous values and Z-scores (age) showed statistically significant but weak correlations with DXA measurements, whereas correlation coefficients between DXA *Z*-scores and pQUS Z-scores (height) revealed to be non-significant. This is in accordance with the reference data provided by Barkmann et al. (2002), who found that the QUS signals correlated less with height compared to age. Therefore the Z-scores (height) were left out for further analysis.

Our analysis showed different results for boys and girls. Only boys had statistically significant correlations for continuous values as well as Z-scores when comparing pQUS and DXA. This is in agreement with the study of Halaba et al. (2005), who found a significant correlation between QUS and DXA in boys (0.40–0.47, p = 0.000) and no correlation in girls. They evaluated 150 healthy Caucasian patients aged from 14 to 19 years. According to Halaba et al., the gender-related bone differences could be related to puberty development and influence of bone size as a result of earlier skeletal maturation in girls compared to boys. Our children had a lower age range and were mainly prepubertal. We do not have an explanation for this phenomenon and therefore suggest that these gender differences should be further investigated.

The association between QUS and DXA has previously been evaluated in a number of studies (Gianni et al., 2008; Tuna et al., 2008; Van Rijn et al., 2000; Di Mase et al., 2012; Falcini et al., 2000; Gonçalves et al., 2014; Hartman et al., 2004a; Pluskiewicz et al., 2002; Sani et al., 2011; Sundberg et al., 1998; Xu et al., 2014; Bąk-Drabik et al., 2016; Catalano et al., 2017; Olszynski et al., 2016; Zuckerman-Levin et al., 2007; Mora et al., 2009; Weeks et al., 2016; Halaba et al., 2005; Chong et al., 2015; Christoforidis et al., 2010; Christoforidis et al., 2011; Hartman et al., 2004b; Williams et al., 2012; De Schepper et al., 2012; Catalano et al., 2013). Table 6 presents an overview of these studies. The results are inconsistent and difficult to compare to our study, partly because a number of studies investigated different populations or used different measurement sites of QUS. Seven studies used the same equipment as we did, comparing pQUS to DXA (Di Mase et al., 2012; Gonçalves et al., 2014; Pluskiewicz et al., 2002; Bąk-Drabik et al., 2016; Catalano et al., 2017; Halaba et al., 2005; Catalano et al., 2013). The correlation coefficients between pQUS and DXA found by Pluskiewicz et al. (2002) and Di Mase et al. (2012) are in agreement with our calculations (0.45–0.56 and 0.42–0,52, respectively). As mentioned above, Halaba et al. (2005) found positive correlations only in boys using continuous values, comparable to our study. In contrast to our results, Halaba et al. found no correlation for both sexes using the *Z*-scores. Nevertheless, in agreement with our analyses these studies found comparable poor correlation coefficients and thereby questioning the equivalence of pQUS and DXA. In contrast, two other studies found relatively stronger correlations for the continuous values (0.59–0.74, p < 0.05), but no significant correlations while comparing Z-scores, except for the AD-SoS Z-score and whole body Z-score which had a poor significant correlation coefficient (0.31, p < 0.02) (Gonçalves et al., 2014; Bąk-Drabik et al., 2016). These five studies evaluated children and adults at an age ranging from 4 to 27 years, while Catalano et al., 2013, Catalano et al., 2017 evaluated postmenopausal women. The younger and smaller age range in our group could be an explanation for the different results we found.

**Table 6:** Literature study of studies investigating the association between the measurements of DXA and QUS.

Author (yr)	QUS	Age (yr)	Sample size	Morbidity	Statistical method	Outcome		Correlation coefficients (r)	Authors' conclusion
						QUS	DXA		
Pluskiewicz et al. (2002)	Phalanges	9–23	30	ESRF	LCC, SCC	AD-SoS	LS/TB BMD, *Z*-scores	0.45–0.56	+
Halaba et al. (2005)	Phalanges	14–19	150	Healthy	LRA, MRA	AD-SoS, *Z*-score	LS/TB BMD, TB BMC, Z-scores	M: 0.40–0.47, F/Z-scores: NS	−
Di Mase et al. (2012)	Phalanges	4–18	25	Idiopathic SH	PCC, MRA	AD-SoS, BTT, *Z*-scores	LS BMD, Z-score	0.42–0.52	+
Catalano et al. (2013)	Phalanges	43–78	80	Postmenopause	SCC	AD-SoS, BTT, UBPI, T-scores	FN BMD T-score, LS BMD T-score	FN T-score: 0.61, LS T-score: 0.55	+
Gonçalves et al. (2014)	Phalanges	6–27	70	21-OHD	PCC, SCC, CT, ROC	AD-SoS, BTT, UBPI, *Z*-scores	LS/TB BMD, Z-scores	0.59–0.72, Z-scores: no correlation	+/−
Bąk-Drabik et al. (2016)	Phalanges	10–15	51	IBD	PCC, SCC	AD-SoS, Z-score	LS/TB BMD, *Z*-scores	Continuous: 0.69–0.74, TB *Z*-scores: 0.31, LS Z-scores: NS	−
Catalano et al. (2017)	Phalanges	60–70	60	Postmenopause, BC	SCC, MRA	AD-SoS, BTT, UBPI	LS/FN BMD	AD-SoS/FN BMD: 0.32	+
Olszynski et al. (2016)	Radius/tibia/phalanges	30–97	4123	Healthy	PCC	SOS	FN, hip, LS BMD	0.21–0.29	−
Hartman et al. (2004a)	Radius/tibia	4–18	40	CRD	PCC, LRA	SOS, Z-score	LS BMD, Z-score	Radius: 0.54, Tibia: NS	+
Hartman et al. (2004b)	Radius/tibia	5–18	41	Celiac disease	LRA, MRA	SOS, Z-score	LS BMD, Z-score	0.3	−
Zuckerman-Levin et al. (2007)	Radius/tibia	11–39	27	Turner's syndrome	PCC	SOS, Z-score	LS/FN/TB BMD, Z-scores	0.46–0.63	−
Gianni et al. (2008)	Radius/tibia	4–6	45	Ex-preterm	PCC	SOS, Z-score	LS BMD, Z-score	NS	−
Mora et al. (2009)	Radius/tibia	4–22	88	HIV-infection	MRA, ×^2^ test	SOS, Z-score	LS/TB BMD/BMC, Z-scores	0.58–0.66	+
Christoforidis et al. (2010)	Radius/tibia	4–18	27	Hemophilia A	PCC, Cк	SOS, Z-score	LS BMD, Z-score	Tibia: NS, Radius: −0.019	−
Christoforidis et al. (2011)	Radius/tibia	2–16	20	CKD	PCC, SCC, MRA	SOS, Z-score	LS BMD, Z-scores	Tibia: 0.129, Radius: 0.022	−
Williams et al. (2012)	Radius/tibia	5–20	621	Healthy, CF, obese	CT, BAA	SOS, Z-score	LS BMD, BMAD, Z-scores	−	−
De Schepper et al. (2012)	Radius	12–38	64	CF	LRA, MRA	SOS, Z-score	TB BMC/BMD, Z-scores	0.39–0.44, Z-scores: NS	−
Chong et al. (2015)	Radius	7–11	134	Healthy	CT, BAA, ROC	SOS, Z-score	TB BMD, Z-score	−	−
Van Rijn et al. (2000)	Tibia	7–23	146	Healthy	MRA	SOS	LS/TB BMD, BMAD	0.63–0.81	+
Tuna et al. (2008)	Tibia	40–72	200	Postmenopause	LRA, SCC, CT, ROC	SOS	LS/FN BMD	0.29–0.36	−
Sundberg et al. (1998)	Calcaneus	11–16	280	Healthy	PCC	SOS, BUA, SI	TB, LS, FN BMD	0.44–0.70	−
Falcini et al. (2000)	Calcaneus	6–18	53	Juvenile arthritis	PCC, LRA	BUA, *Z*-score	LS BMD, Z-score	0.83	+
Sani et al. (2011)	Calcaneus	6–17	8	Thalassemia	PCC	SOS, BUA	TB/LS BMD	BUA: 0.492–0.507, SOS: NS	+/−
Xu et al. (2014)	Calcaneus	5–19	392	Healthy	PCC	SOS, BUA, SI	TB BMC/BMD	0.690–0.693	+
Weeks et al. (2016)	Calcaneus	4–18	389	Healthy	PCC, LRA, MRA	BUA	TB/LS/FN BMD, BMC	0.47–0.56	− to +/−

*Morbidity*: ESRF, End-stage renal failure; Idiopathic SH, Idiopathic subclinical hypothyroidism; BC: breast cancer; CRD, Chronic rheumatic disease; IBD, Inflammatory bowel disease; CF, Cystic fibrosis; 21-OHD, 21-hydroxylase deficiency; CKD, Chronic kidney disease.

*Statistical methods*: PCC, Pearson's correlation coefficient; LCC, linear correlation coefficient; SCC, Spearman's correlation coefficient; CT, cross tabulation; ROC, receiver operating characteristic analysis; LRA, linear regression analysis; MRA, multiple regression analysis; BAA, Bland-Altman analysis; Cк, Cohen к analysis.

*Outcome*: LS, lumbar spine; TB, total body; FN, femoral neck; AD-SoS, amplitude-dependent speed of sound; SOS, speed of sound; BTT, bone transit time; UBPI, ultrasound bone profile index; BUA, broadband ultrasound attenuation; SI, stiffness index; BMC, bone mineral content; BMD, bone mineral density; BMAD, bone mineral apparent density; NS, not significant; F, female; M, male.

*Author*'*s conclusion*: +, good association; +/−, moderate association; −, weak/poor association.

Furthermore, Table 6 gives an overview of the authors' conclusion per study, showing different interpretations for in general a relatively low correlation between QUS and DXA. An advantage of our study was that, besides the small range of age of the participants, more statistical tests were used to compare both techniques, leading to a more reliable overall conclusion.

The partial correlation coefficient was used to evaluate the effect of possible confounders on the original correlation. To our knowledge only a few studies that evaluated the association between pQUS and DXA looked at the influence of possible confounding factors with regard to correlation coefficients. Halaba et al. (2005) and Di Mase et al. (2012) performed a multiple linear regression analysis to evaluate the effect of anthropometric characteristics on DEXA or QUS measurements using confounders such as age, sex, weight, height and BMI.

Only four studies used other statistical tests such as the Bland-Altman analysis (Chong et al., 2015; Williams et al., 2012) and cross tabulation (Tuna et al., 2008; Gonçalves et al., 2014; Chong et al., 2015; Williams et al., 2012). Although these studies looked at tibial and radial QUS, their conclusions are consistent with our findings of poor agreement between the two techniques and a large discrepancy in differentiating the same children as having normal or reduced bone mineralization.

The overall absence of an association between DXA and pQUS for continuous values and *Z*-scores could be explained by the fact that measurements were influenced by different bone composition and bone mineralization at different sites. In addition, children may be in different growth phases and some bones may grow faster than others, which potentially could have an effect on the bone development and thereby may have affected the results. Quantitative ultrasound can be applied on various parts of the extremities, such as the phalanx, radius, tibia and calcaneus. Recent studies suggest that the phalanges may be the most appropriate measurement site, because this site is sensitive to changes in bone status (Takada et al., 1997a; Ventura et al., 1996; Takada et al., 1997b).

For the assessment of pQUS we chose the DXA scan as the golden standard for comparison. Quantitative Computed Tomography (QCT), or peripheral QCT may have been good alternatives and may have provided more accurate results and additional information on bone strength (Pezzuti et al., 2017). However, QCT uses a relatively high radiation dose, especially for young children in research settings, and for both methods normative data for a pediatric population are lacking (Pezzuti et al., 2017; Fonseca et al., 2013). DXA has been recommended as an appropriate method for clinical densitometry of infants and young children by the International Society for Clinical Densitometry (ISCD) in 2013 (Gordon et al., 2013). The advantage of QUS is the ability to easily repeat measurements, giving the opportunity to follow the development of bone over time, especially under circumstances were diagnostic tools as DXA are not possible or available. Our results were limited to a single measurement per child. Therefore intra-individual repeated measurements with QUS should be further evaluated with regard to the reliability to predict the intra-individual development of bone over a longer time period.

Our study had several limitations. The number of patients was limited. Firstly, this limited the number of confounders we were able to use for calculation of the partial correlation coefficient. Secondly, low bone mineralization with a significant *Z*-score below 2.0 SD was only found in 2 out of 60 children for lumbar spine measurements, and none for the whole body measurements. To increase the number of children with deviant bone mineralization we chose a cut-off value of −1.0 SDS for determination of normal or low bone mineralization, in contrast to general practice. This is in accordance with the study of Gianni et al., who evaluated the prevalence of *Z*-scores <−1.0 SDS for tibial or radial QUS (Gianni et al., 2008), because the QUS values were higher in comparison with DXA. According to the literature a similar cut-off value can be used for AD-SoS and BTT (Di Mase et al., 2012; Baroncelli et al., 2006).

In general, the Z-scores were based on reference data derived from different populations. The reference data of the DXA Z-scores were based on an U.S. population, while the reference data for the pQUS Z-scores were recorded from an Italian population. It is not known whether the populations are comparable to our population and theoretically this might explain some of our differences.

## 5 Conclusion

This study demonstrated a weak association between DXA and pQUS measurements, established from a statistically significant weak correlation, a poor agreement and a discrepancy in differentiating the same children with normal or reduced bone mineralization. Therefore, pQUS measurements are not equivalent to DXA for the evaluation of bone health and cannot replace the DXA in former preterm born children at the age of 9 to 10 years.
